# Differential microglial dynamics and neuroinflammation underlying neuropathic pain in the central nervous system: comparative insights from spinal cord injury and compressive myelopathy models

**DOI:** 10.3389/fncel.2026.1769004

**Published:** 2026-01-23

**Authors:** Arisa Kubota, Hideaki Nakajima, Kazuya Honjoh, Shuji Watanabe, Ai Takahashi, Akihiko Matsumine

**Affiliations:** Department of Orthopaedics and Rehabilitation Medicine, Faculty of Medical Sciences, University of Fukui, Fukui, Japan

**Keywords:** central nervous system, degenerative compressive myelopathy, microglia/macrophage, neuroinflammation, neuropathic pain, ossification of posterior longitudinal ligament, spinal cord, spinal cord injury

## Abstract

**Introduction:**

Neuropathic pain (NeP) is a major complication of spinal cord disorders that is refractory to therapy and impairs quality of life. Acute neuroinflammatory responses occur after spinal cord injury (SCI), but chronic-phase microglia/macrophage (M/M) dynamics and their involvement in degenerative compressive myelopathy (DCM) are unclear. Brain M/M may contribute to persistent NeP; however, comparative analyses of SCI and DCM are lacking. The aim of this study was to investigate M/M activation and pain-related signaling dynamics in the spinal cord and brain, and their roles in chronic NeP following SCI and DCM.

**Methods:**

Contusive SCI was induced in C57BL/6N mice. Chronic compression was modeled using *ttw*/*ttw* mice. Motor function was assessed using the Basso Mouse Locomotor Scale. Mechanical and thermal sensitivities were evaluated. M/M activation and pain-related molecules (p-p38, p-ERK1/2) were assessed in spinal and brain regions using immunohistochemical staining. Cytokine expression was analyzed using western blotting.

**Results:**

SCI mice showed early M/M activation at the injured site with spread to the lumbar enlargement, paralleling mechanical and thermal hypersensitivity. In DCM, M/M activation increased with compression severity, but did not extend to the lumbar enlargement. Both models showed M/M and pain-related upregulation of molecules in the hippocampus and amygdala, and thalamic activation in acute SCI or moderate-to-severe compression. Pro-inflammatory cytokines peaked acutely in SCI and under moderate compression in DCM. Anti-inflammatory cytokine induction was limited in DCM.

**Discussion:**

Distinct neuroinflammatory patterns underlie chronic NeP in SCI and DCM. SCI shows M/M activation shifting from the injured site to the lumbar enlargement and limbic brain regions, consistent with chronic below-level pain. DCM shows localized M/M activation, but earlier hippocampal/amygdalar involvement, consistent with chronic at-level pain. These findings suggest pathology-specific therapeutic windows for targeting M/M-mediated neuroinflammation to prevent NeP.

## 1 Introduction

Spinal cord disorders, including acute spinal cord injury (SCI) and degenerative compressive myelopathy (DCM) such as cervical spondylotic myelopathy (CSM) and ossification of the posterior longitudinal ligament (OPLL), are significant medical challenges owing to their association with motor impairment and intractable chronic neuropathic pain (NeP). Despite advances in surgical and pharmacological interventions, the persistence of such symptoms diminishes quality of life and daily functioning of patients (Finnerup et al., 2015; Inoue et al., 2017). A study in Japan found that over half of patients (53.3%) with spinal conditions experienced NeP, as determined by a screening questionnaire designed to identify potential NeP cases. The highest rate of NeP was found in patients with CSM (77%), followed by those with OPLL (75%) and SCI (65%) (Yamashita et al., 2014). An epidemiological study indicated that 65.0%–78.8% of patients with NeP have chronic pain (Eggermont et al., 2014). NeP is characterized by heightened pain sensitivity such as allodynia and is refractory to conventional therapy in 50%–70% of cases (Christensen et al., 1996; Nakajima et al., 2019a,b), showing the need for greater understanding of the pathophysiology and identification of new therapeutic targets.

A key pathological mechanism implicated in SCI and DCM is neuroinflammation driven by prolonged activation of microglia and macrophages (M/M) in the central nervous system (Watanabe et al., 2015; Gwak et al., 2017; Chen et al., 2018; Takeura et al., 2019). M/M polarization into pro-inflammatory (M1) and anti-inflammatory (M2) phenotypes plays a pivotal role in modulating neuronal-glial interactions, thereby influencing neuronal function, tissue repair and pain signaling. The acute and subacute phases of neuroinflammatory responses after SCI have been widely studied, but the dynamics of M/M activity in the chronic phase of SCI remain poorly characterized. Furthermore, little is known about these mechanisms in patients with DCM. Recently, M/M activation in the brain, especially in the amygdala, hippocampus, and thalamus, has been related to chronic NeP (Apkarian et al., 2009; Zhao et al., 2007; Maldonado-Bouchard et al., 2016; Barcelon et al., 2019).

Knowledge of the distribution of M/M in brain-spinal cord lesions in the chronic phase, including differences between those in SCI and DCM, is crucial for comprehending the underlying mechanisms and developing new treatment approaches for NeP in patients with SCI and DCM. Therefore, the aim of this study is to analyze the dynamic changes and differences in M/M in the central nervous system in the brain, injured/compression area, and lumbar enlargement during the acute and chronic phases of SCI and from mild to severe compression in DCM, and their relationship with expression of pain-related molecules.

## 2 Materials and methods

### 2.1 Experimental mouse models

**Figure 1:**
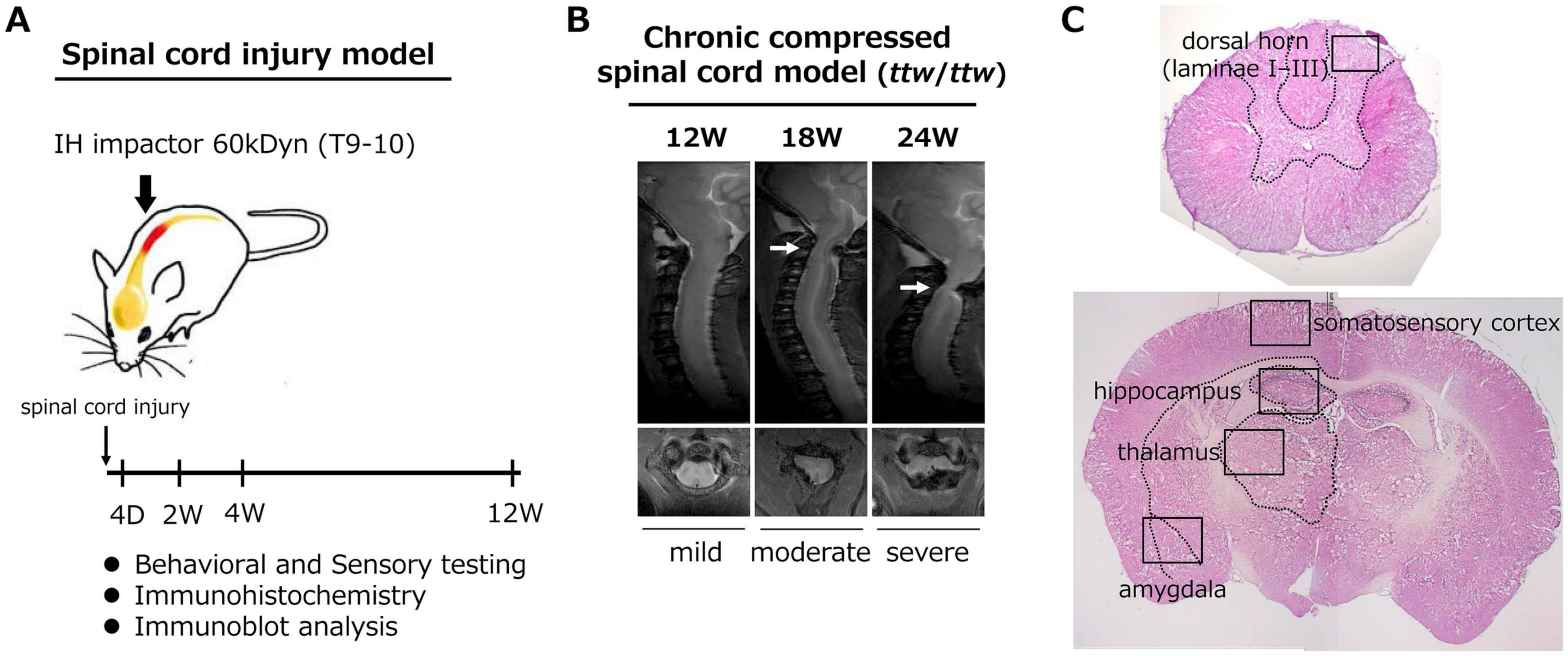
Experimental design and timeline of behavioral, histological, and molecular analyses in the spinal cord injury (SCI) model **(A)** and chronic compressed spinal cord (DCM) (*ttw*/*ttw*) model **(B)**. **(C)** Assessment regions in the spinal cord and brain.

In the acute SCI model, adult male C57BL/6N mice (10–12 weeks, male, Nihon SLC, Shizuoka, Japan) were subjected to contusion injury at T9–T10 using an Infinite Horizon Impactor (60 kdyn) or sham-operated by laminectomy only, as described previously (Figure 1A) (Scheff et al., 2003). For the DCM model, *ttw*/*ttw* mice (12–24 weeks, male, Central Institute for Experimental Animals, Kawasaki, Japan) were used, with age-matched male Institute of Cancer Research (ICR) mice as controls. Calcified masses at C1-C2 cause progressive spinal cord compression and motor deficits, and often produce extensive motor weakness at age 18–24 weeks (Figure 1B) (Nakajima et al., 2020b; Takeura et al., 2019; Uchida et al., 2012). In the SCI model, the acute phase was defined as 0–4 days after injury, the subacute phase as 5–14 days, and the chronic phase as ≥28 days, in accordance with commonly used experimental classifications of SCI (Crown et al., 2006; Hulsebosch, 2008). In addition, to examine longer-term pathological and behavioral changes, an extended chronic phase at 12 weeks after injury was also included in the present study. In the DCM model, according to previous papers that analyzed the compressed transverse remnant area of the spinal cord (TRANS%) using transaxial serial sections (Uchida et al., 2012), *ttw*/*ttw* mice were divided into three groups: mild (12 weeks, TRAS% > 50), moderate (18 weeks, TRAS% between 50% and 70%), and severe (24 weeks, TRAS% ≤ 50%) compression group in this study. Because the extent of spinal cord compression varies among individual *ttw*/*ttw* mice, animals whose TRANS% values did not meet the predefined criteria for each severity category were excluded from the analysis to ensure consistent classification of compression severity. The mice were maintained in an isothermic cage and housed under a 12-h light–dark cycle in a bacteria-free biologically clean room with *ad libitum* access to food and water.

### 2.2 Behavioral and sensory testing

Locomotor recovery was assessed using the Basso Mouse Locomotor Scale (BMS, 0–9) for the SCI model (*n* = 5 mice at each time point) (Basso et al., 2006). In *ttw*/*ttw* mice, motor scoring was not feasible due to joint contracture (Takeura et al., 2019). Mechanical allodynia was tested using a Dynamic Plantar Aesthesiometer (Ugo Basile, Comerio, Italy) (Martucci et al., 2008). Thermal sensitivity was tested as previously described (*n* = 5 mice at each time point) (Watanabe et al., 2015; Hargreaves et al., 1988; Hoschouer et al., 2010). Two independent examiners who were blinded to the experimental results tested the mice.

### 2.3 Immunohistochemistry

The spinal cord and brain were carefully dissected and fixed. Immunohistochemical staining was performed on serial 20-μm axial or coronal frozen sections (*n* = 5 mice at each time point). Tissue blocks were stained immunohistochemically, as described previously (Nakajima et al., 2020a,b; Takeura et al., 2019; Watanabe et al., 2015). The primary antibodies (Abs) used were rabbit anti-phospho-p38 MAP kinase (p-p38) polyclonal Ab (1:400, Cell Signaling Technology, Beverly, MA); rabbit anti- p44/42 MAPK (p-ERK1/2) polyclonal Ab (Cell Signaling Technology); and rabbit anti-CD11b Ab (1:50, Abcam, Cambridge, UK). Sections were incubated with Alexa Fluor-conjugated 488 or 568 secondary antibodies (dilution, 1:250, Molecular Probes, Eugene, OR) and examined under a fluorescence microscope (Olympus AX80, 200 Olympus Optical, Tokyo, Japan) using the 488- and 543-nm lines of the argon/helium-neon laser.

### 2.4 Semi-quantitative analysis of tissue staining

Semi-quantitative analysis of dorsal horn neuronal hyperexcitability was performed at the lesion site (injured/compressed site and lumbar enlargement) and target brain regions related to pain modulation (amygdala, hippocampus, thalamus, and somatosensory cortex) identified using anatomical landmarks and the Paxinos and Franklin (2004) mouse brain atlas (Figure 1C) (Apkarian et al., 2009). The hippocampus and amygdala were selected *a priori* as representative limbic regions involved in affective and cognitive dimensions of pain, and the present supraspinal analysis was therefore designed to target sensory–affective pain domains rather than to comprehensively survey all canonical pain modulatory regions. For each site, five axial or coronal sections were analyzed with 20 high-magnification images (400×) per section. Fields were selected in a non-overlapping and systematic manner within predefined anatomical regions based on established landmarks, rather than on signal intensity, to minimize selection bias. All images were acquired using identical microscope settings across experimental groups. Quantitative measurements obtained from multiple fields and sections were averaged within each animal, which was defined as the experimental unit. Accordingly, no individual image or section was treated as an independent replicate, thereby avoiding pseudoreplication. Only CD11b-positive signals colocalized with DAPI-positive nuclei were included in the analysis; isolated or fragmented CD11b signals lacking nuclear staining were excluded. The numbers of CD11b-positive cells and those co-expressing p-p38 MAPK or p-ERK1/2 in laminae I–III of the dorsal horn and each brain region were counted. All counting were performed in triplicate by each of two observers and the average value was used.

### 2.5 Immunoblot analysis

For immunoblot analysis, spinal cord tissue was collected from the SCI and DCM models (*n* = 3 mice at each time point) and stored at −80 °C. Protein samples were prepared, separated by 12.5% SDS-PAGE, transferred to PVDF membranes, and blocked as described previously (Takeura et al., 2019; Watanabe et al., 2015). The membranes were incubated overnight at 4 °C with the following primary Abs: anti-TNF alpha Ab (1 μg/mL, Abcam), anti-IL-12 Ab (1:1,000, Santa Cruz Biotechnology, Dallas, TX), anti-IL-4 Ab (1:1,000, Santa Cruz Biotechnology), and anti-IL-10 Ab (1:1,000, Proteintech, Chicago, IL). After washing, the membranes were incubated with the respective HRP-conjugated secondary Abs (1:1,000, Santa Cruz Biotechnology). Protein bands were identified using the Western Lightning ECL Pro system (PerkinElmer) and their intensity was quantified with Image J (National Institutes of Health, Bethesda, MD), normalized to β-actin. Precision Plus Protein Western Standards (Bio-Rad) were used as molecular weight markers.

### 2.6 Statistical analysis

Data are presented as mean ± SD. Repeated behavioral measurements across multiple time points were analyzed using repeated-measures ANOVA or linear mixed-effects models, as appropriate, to account for within-subject correlations. For histological and immunohistochemical analyses, the individual animal was treated as the experimental unit, and values from multiple sections or fields were averaged per animal prior to analysis. Multiple comparisons were adjusted using the Benjamini–Hochberg false discovery rate procedure. A two-sided adjusted *p* value < 0.05 was considered statistically significant. All statistical analyses were conducted using EZR (Saitama Medical Center, Jichi Medical University, Saitama, Japan), which serves as a graphical user interface for R (The R Foundation for Statistical Computing, Vienna, Austria) (Kanda, 2013).

## 3 Results

### 3.1 Locomotor function and pain hypersensitivity to mechanical and thermal stimulation

**Figure 2:**
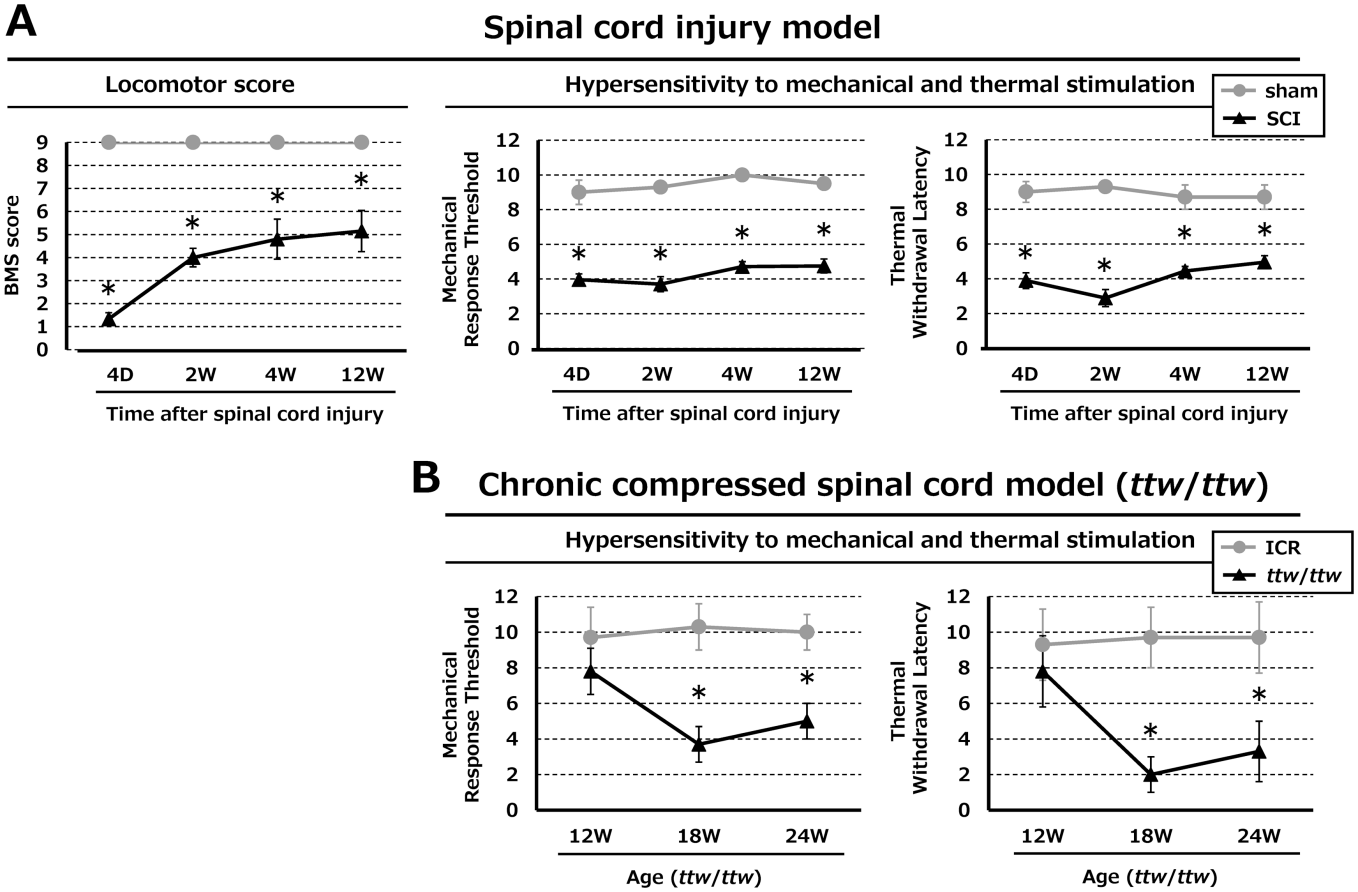
Locomotor function and pain hypersensitivity in the SCI and DCM models. **(A)** SCI induced deterioration of hind-limb motor function and hypersensitivities to mechanical and thermal stimulation. **(B)** Chronic compression of the spinal cord was associated with hypersensitivity to mechanical and thermal stimulation. *p < 0.05.

In the mouse model of contusion SCI, hindlimb motor function deteriorated until reaching a stable level 4 weeks after injury, followed by some improvement thereafter (Figure 2A). Thresholds for mechanical response and thermal sensitivity were reduced, with the most intense pain hypersensitivity occurring 2 weeks post-SCI. In *ttw*/*ttw* mice, the mechanical and thermal sensitivity scores were lower than those of ICR mice at ages 18 and 24 weeks (Figure 2B). Assessment of locomotor function in *ttw*/*ttw* mice was not possible because joint contracture progression over time.

### 3.2 Distribution of microglia/macrophages in the dorsal horn

**Figure 3:**
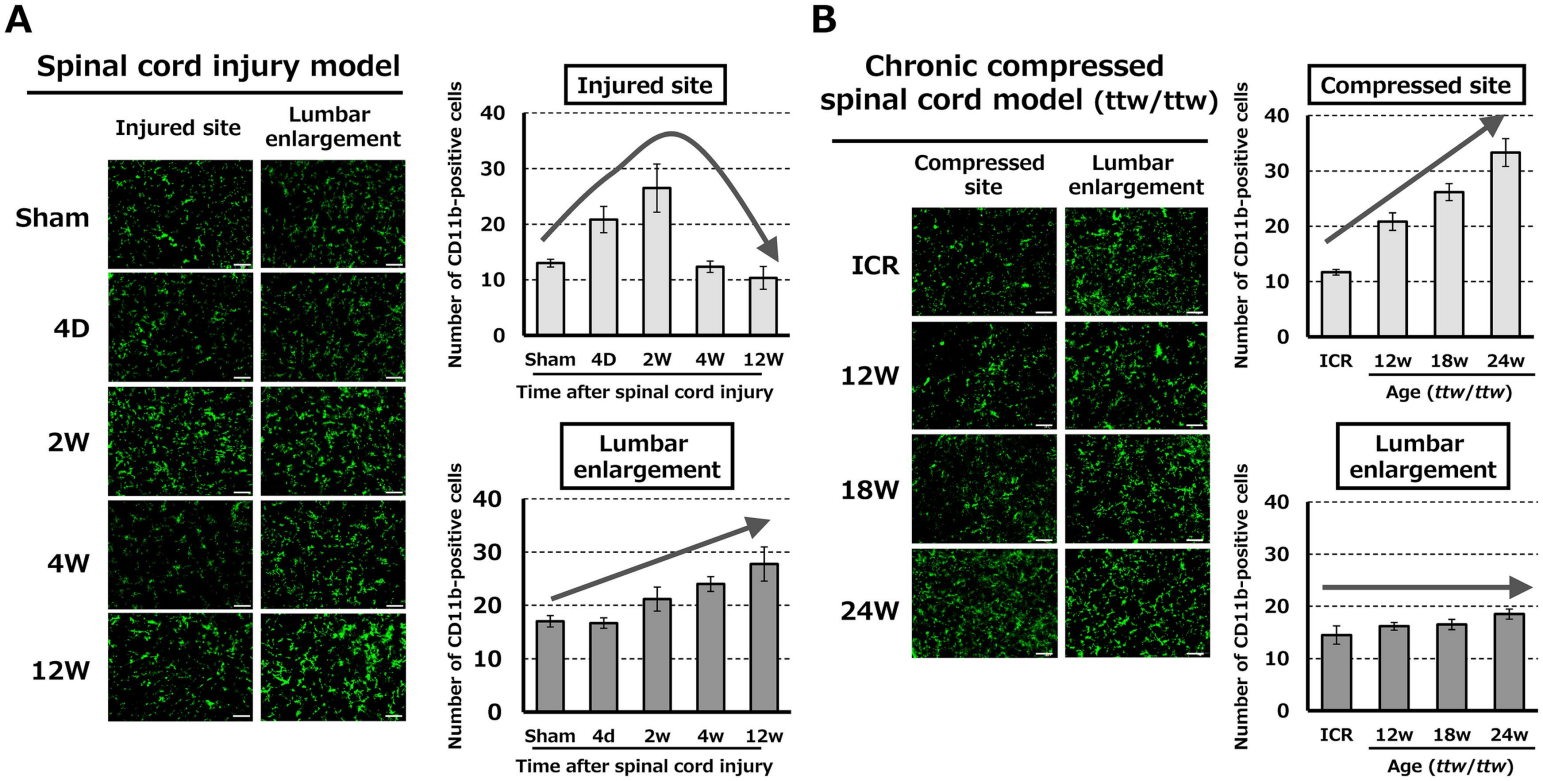
Distribution of CD11b-positive microglia/macrophages in the spinal cord. **(A)** In the SCI model, CD11b-positive cells at the lesion site peaked at day 14 and declined thereafter, while cell numbers at the lumbar enlargement progressively increased during the chronic phase. Scale bars = 50 μm. **(B)** In the DCM model, the number of CD11b-positive cells at the compression site increased in proportion to the severity of compression (notably at 18 and 24 weeks), whereas there were no significant changes in the lumbar enlargement. Scale bars = 50 μm.

In the SCI model, the number of CD11b-positive cells at the injured site peaked at 14 days and decreased in the chronic phase after SCI. At the lumbar enlargement, CD11b-positive cells increased progressively during the chronic phase after SCI (Figure 3A; Supplementary Figure S1A). In contrast, in the DCM model (*ttw*/*ttw*), CD11b-positive cells at the injured site increased in proportion to the degree of spinal cord compression, especially in mice aged 18–24 weeks. There were no significant changes in CD11b-positive cells in the lumbar enlargement with increasing degrees of compression of the cervical spinal cord (Figure 3B; Supplementary Figure S1B).

**Figure 4:**
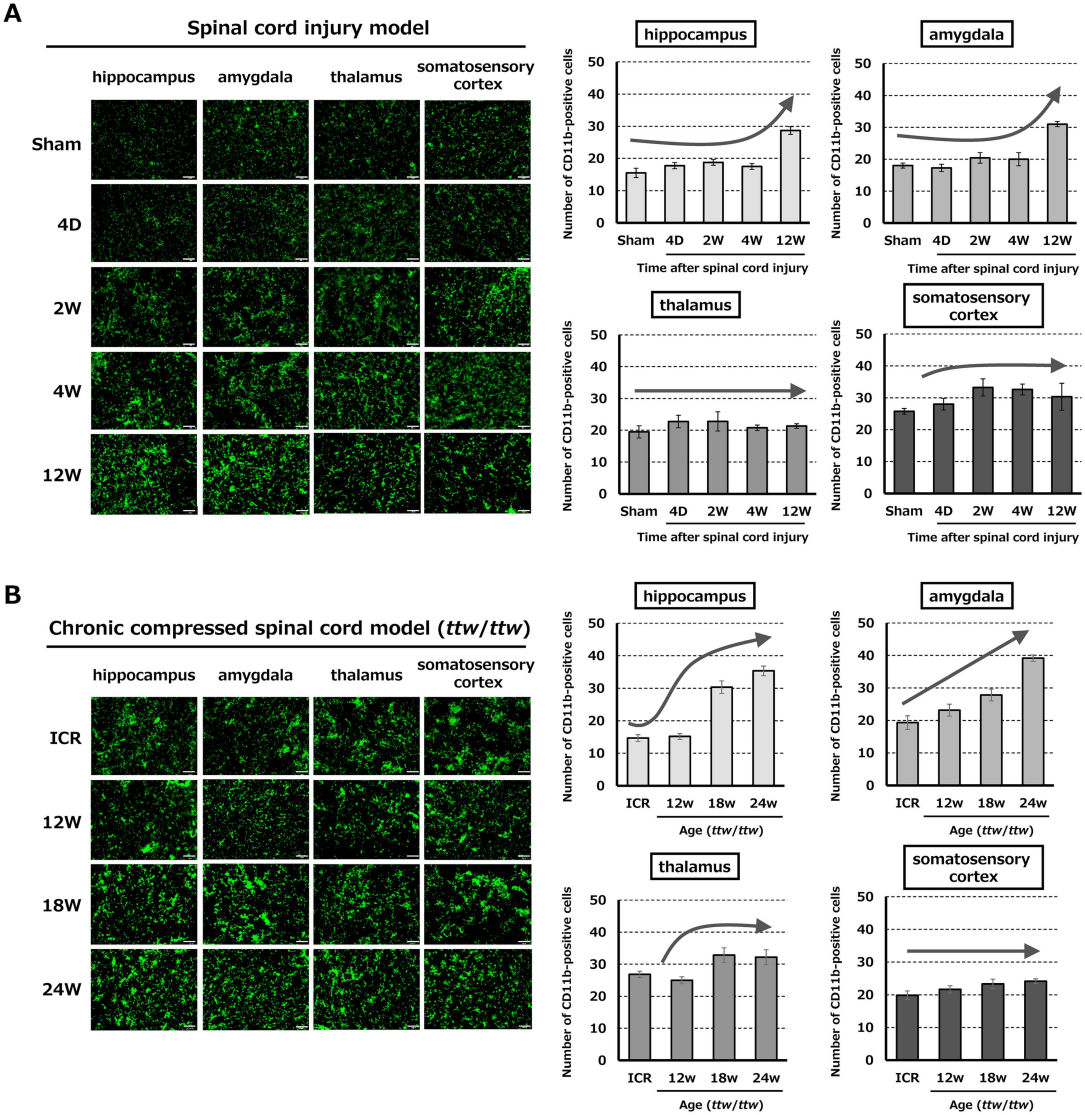
Microglia/macrophage activation in the brain in the SCI and DCM models. **(A)** In SCI, CD11b-positive cells significantly increased in the hippocampus and amygdala at 12 weeks, while there were no changes in the thalamus or somatosensory cortex. Scale bars = 50 μm. **(B)** In the DCM model, CD11b-positive cells increased in the hippocampus and amygdala of *ttw*/*ttw* mice in proportion to the severity of compression. Significant increases were also detected in the thalamus of 18- and 24-week-old mice, but not in the somatosensory cortex. Scale bars = 50 μm.

Changes in CD11b-positive cells in the brain are shown in Figure 4. In the SCI model, CD11b-positive cells in the hippocampus and amygdala significantly increased 12 weeks after SCI, whereas these cells did not change significantly in the thalamus and somatosensory cortex (Figure 4A; Supplementary Figure S2A). In the DCM model, CD11b-positive cells in the hippocampus and amygdala increased with the degree of spinal cord compression. CD11b-positive cells also increased in the thalamus of mice aged 18–24 weeks compared to 12 weeks. There were no significant changes in the somatosensory cortex (Figure 4B; Supplementary Figure S2B).

### 3.3 Changes in expression of pain-related molecules

**Figure 5:**
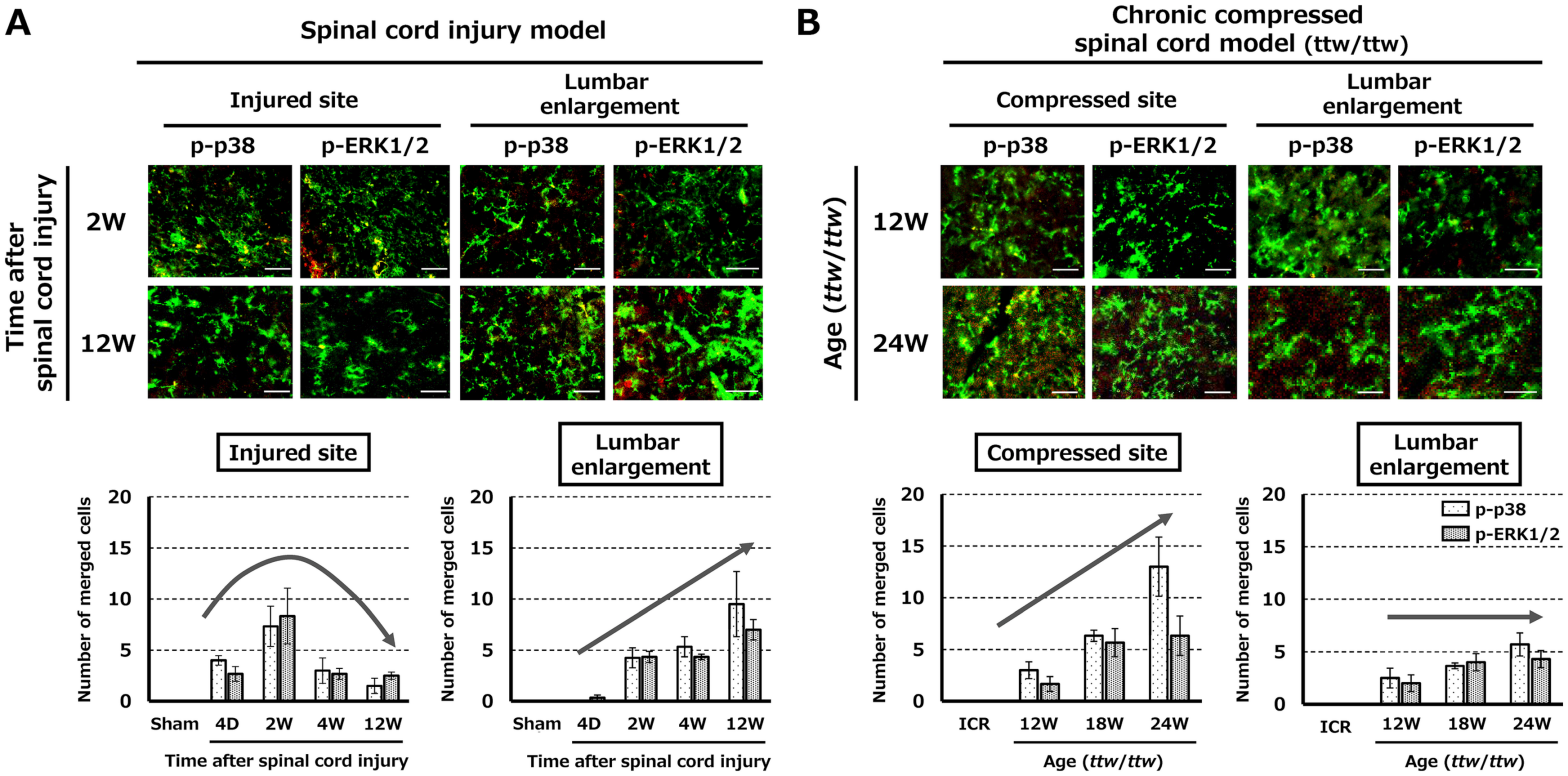
Expression of pain-related molecules in the spinal cord. **(A)** In the SCI model, p-p38 and p-ERK1/2 expression colocalized with CD11b at the lesion site peaked at 2 weeks post-injury and increased in the lumbar enlargement during the chronic phase (12 weeks). Scale bars = 50 μm. **(B)** In the DCM model, expression of p-p38 and p-ERK1/2 colocalized with CD11b progressively increased at the compression site, with little change in the lumbar enlargement. Scale bars = 50 μm.

Expression of p-p38 and p-ERK1/2 colocalized with CD11b in the injured/compressed spinal cord and lumbar enlargement in the SCI and DCM models in response to changes in CD11b-positive cells over time. In the SCI model, expression of p-p38 and p-ERK1/2 colocalized with CD11b in the injured site peaked at 2 weeks after injury and was significantly higher in the lumbar region through the chronic phase at 12 weeks after injury (Figure 5A; Supplementary Figure S3A). In the compressed site of the DCM model, expression of p-p38 and p-ERK1/2 colocalized with CD11b increased with the degree of spinal cord compression, but there was little change in the lumbar enlargement (Figure 5B; Supplementary Figure S3B).

**Figure 6:**
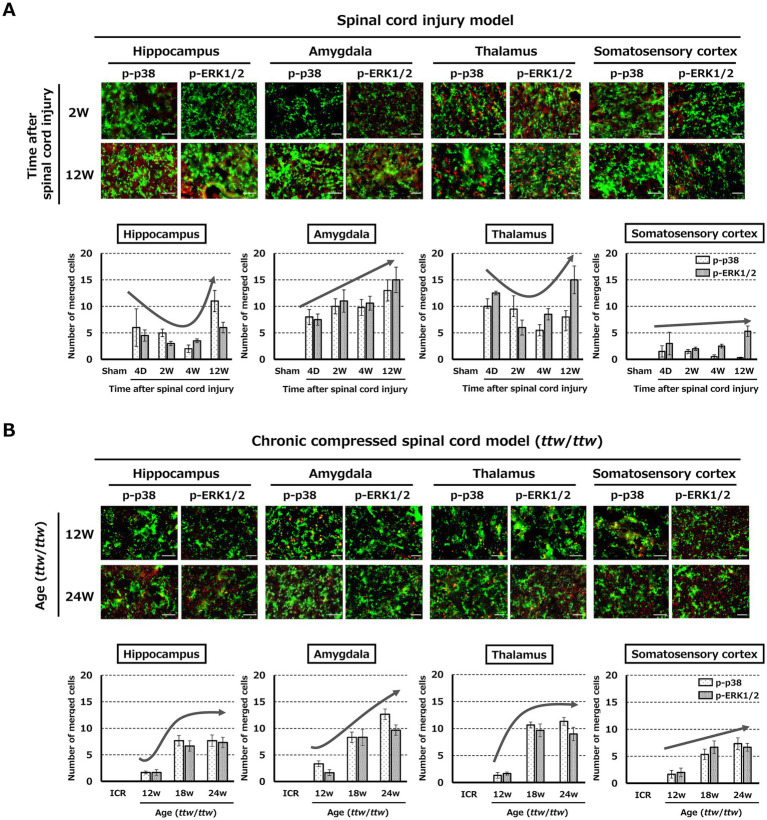
Expression of pain-related molecules in the brain. **(A)** In the SCI model, there was significant expression of p-p38 and p-ERK1/2 colocalized with CD11b in the hippocampus and amygdala during the chronic phase (12 weeks) and in the thalamus at both acute and chronic time points. Scale bars = 50 μm. **(B)** In the DCM model, expression of pain-related molecules increased in the hippocampus, amygdala, and thalamus under moderate compression (18 weeks). Scale bars = 50 μm.

In contrast, in the brain, expression of p-p38 and p-ERK1/2 colocalized with CD11b did not necessarily coincide with changes in CD11b expression. In the SCI model, significant expression was observed in the hippocampus and amygdala in the chronic phase at 12 weeks after injury and in the thalamus in the acute (4 days and 2 weeks after injury) and chronic (12 weeks after injury) phases (Figure 6A; Supplementary Figure S4A). In the DCM model, there was increased expression in the hippocampus, amygdala, and thalamus of mice aged 18 weeks with moderate spinal cord compression (Figure 6B; Supplementary Figure S4B). In the somatosensory cortex, there was little expression of p-p38 and p-ERK1/2 colocalized with CD11b in both models.

### 3.4 Expression of pro- and anti-inflammatory cytokines

**Figure 7:**
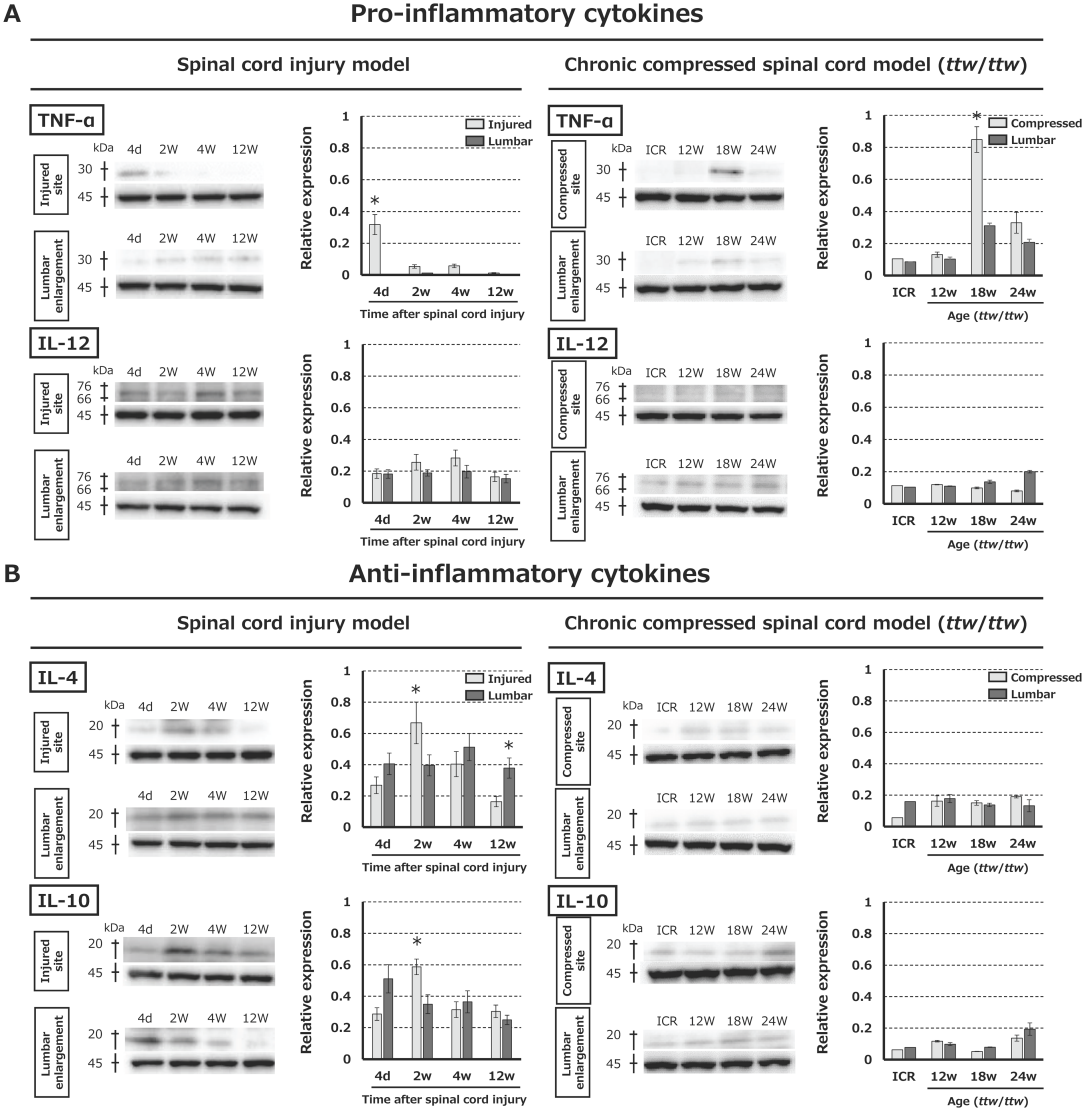
Cytokine expression in the spinal cord in the SCI and DCM models. **(A)** For pro-inflammatory cytokines, TNF-α peaked at 4 days post-injury in SCI and at 18 weeks in DCM, while IL-12 expression did not change significantly in either model. **(B)** Anti-inflammatory cytokines IL-4 and IL-10 peaked at 2 weeks post-injury in SCI, but showed no significant changes in DCM. *p < 0.05.

Western blot analysis was used to assess the levels of pro-inflammatory (TNF-α and IL-12) and anti-inflammatory (IL-4 and IL-10) cytokines at the site of injury and the lumbar enlargement in the SCI and DCM models. At the injured site after SCI, TNF-α peaked at 4 days (Figure 7A), and IL-4 and IL-10 peaked at 2 weeks (Figure 7B). In the DCM model, TNF-α peaked in *ttw*/*ttw* mice aged 18 weeks (Figure 7A). There were no significant changes in anti-inflammatory cytokines (IL-4 and IL-10) at either site in the DCM model (Figure 7B) and no significant changes in IL-12 levels in either model.

## 4 Discussion

NeP is a major unmet clinical problem in patients with spinal cord disorders due to its refractory nature and impact on prognosis and quality of life. Although the epidemiology and clinical prevalence of NeP in SCI and DCM have been well described, the mechanisms underlying their persistence and heterogeneity remain unclear.

Inflammatory responses and M/M play crucial roles in NeP following SCI (Detloff et al., 2008; Hains and Waxman, 2006; Hulsebosch, 2008), and microglial activation can extend caudally to the lumbar enlargement after thoracic SCI (Nakajima et al., 2020a). Such activation in the lumbar enlargement has been associated with below-level pain (Crown et al., 2006; Nesic et al., 2005). These findings show the importance of events at the lesion site and remote microglial changes in the lumbar enlargement. For example, studies have shown that the M1 phenotype of M/M increases markedly at the lesion site in the acute phase (1 week post-injury) and then substantially decreases by 4 weeks post-injury (Francos-Quijorna et al., 2016; Kigerl et al., 2009). In contrast, microglial activation and upregulation of p-p38 signaling occur in the lumbar enlargement in the subacute phase (4 weeks post-injury), and pharmacological inactivation of microglia at this site suppresses both p-p38 expression and pain (Detloff et al., 2008; Hains and Waxman, 2006). Previous studies indicates that MAPK signaling, particularly phosphorylation of p38 MAPK and ERK1/2 in the spinal dorsal horn, is closely associated with the development and maintenance of NeP through neuron–glia interactions (Crown et al., 2006; Hulsebosch, 2008). These observations suggest that below-level pain after SCI depends on remote microglial activation, which occurs with a distinct temporal profile from that observed at the lesion site. In this study, we analyzed M/M dynamics, pain-related molecules, and cytokine changes after SCI in the injured site, lumbar enlargement, and brain. At the injured site, TNF-α levels increased immediately post-injury, followed by peak increase in CD11b-positive M/M and pain-related molecules at 2 weeks. IL-4 increased from the subacute phase onward in the lumbar enlargement. In agreement with previous reports, M/M and pain-related molecular activation dominated the injured site in the acute phase, but shifted to the lumbar enlargement in the subacute-to-chronic phases, where it persisted for a prolonged period. Clinically, these patterns are consistent with the observation that post-spinal cord injury pain, particularly in the lower limbs (below-level pain), tends to become chronic (Detloff et al., 2008; Finnerup et al., 2015; Siddall and Loeser, 2001).

We also investigated the dynamics of M/M across phases in a DCM model. Reports of NeP in this pathology are scarce, although we have previously examined the underlying mechanisms using *ttw*/*ttw* mice. Consistent with earlier findings, CD11b-positive cells increased with compression severity, with activation of p-p38 and p-ERK1/2 (Takeura et al., 2019). M/M activation and pain-related molecules at the compression site increased proportionally to the degree of compression, with TNF-α elevated even under moderate compression. In contrast to the SCI model, there was no significant increase in CD11b-positive M/M or pain-related molecules in the lumbar enlargement. In the DCM model, lumbar changes were minimal, indicating local activation. Clinically, these patterns correspond to the tendency for pain associated with DCM to persist chronically, particularly in the upper extremities (at-level pain) (Siddall and Loeser, 2001; Finnerup et al., 2015; Yamashita et al., 2014).

M/M dynamics were further analyzed in the brain in both models because pain perception includes sensory, affective, and cognitive elements. The sensory-discriminative effect is encoded by the primary and secondary somatosensory cortices (S1 and S2), enabling localization and recognition of noxious stimuli (Hsieh et al., 1995; Jones et al., 1991; Qiu et al., 2006). Consistent with this, many patients with chronic pain present with affective disturbances, such as depression (Liu et al., 2017). Neuroinflammation has been linked to these symptoms, with IL-1β implicated in depression induced by chronic stress (Goshen et al., 2008; Koo and Duman, 2008), increased IL-1β production in the amygdala following chronic stress exposure (Porterfield et al., 2012), and TNF-*α* involvement in anxiety and inflammatory pain (Chen et al., 2013). Several studies have also suggested that brain M/M activation plays a key role in NeP after SCI. SCI has been associated with mood disorders and depression and may induce chronic microglial activation across multiple brain regions (Jure and Labombarda, 2017; Luedtke et al., 2014; Maldonado-Bouchard et al., 2016; Wu et al., 2016; Xue et al., 2019). Microglia in the thalamic ventral posterolateral nucleus become activated 2 weeks after SCI and exhibit hypertrophy for 4 weeks (Zhao et al., 2007). Similarly, elevated levels of inflammatory cytokines have been found in the thalamus of allodynic rats after SCI (Detloff et al., 2008). There is also abundant evidence linking peripheral nerve injury-induced NeP to changes in brain microglia. For instance, CD11b-positive cells are increased in the hypothalamus and periaqueductal gray matter after chronic nerve constriction (Takeda et al., 2009; Riazi et al., 2008), and sciatic nerve constriction models show microglial activation and increased BDNF levels in the periaqueductal gray matter and thalamus 14 days after injury (Giardini et al., 2017). In a chronic sciatic nerve compression model, depression-like behaviors and microglial activation with TNF-α upregulation in the medial prefrontal cortex, hippocampus, and amygdala emerged only after 8 weeks, despite allodynia occurring immediately after chronic constriction injury (Barcelon et al., 2019). Thus, chronic microglial activation in late phases contributes to pain and affective disturbances in the brain. In our study, M/M dynamics were studied in the somatosensory cortex, thalamus, amygdala, and hippocampus in the SCI and DCM models. In the SCI model, increased CD11b-positive M/M and levels of pain-related molecules were observed in the hippocampus and amygdala in the chronic phase (12 weeks post-injury), consistent with involvement of brain microglia in chronic NeP after SCI. In the DCM model, M/M activation and pain-related molecules in the hippocampus and amygdala increased with compression severity, whereas significant increases in these molecules in the thalamus and somatosensory cortex only occurred under moderate or severe compression. Compared with SCI, M/M-associated and pain-related molecular changes in the hippocampus and amygdala were more pronounced, suggesting a higher propensity for refractory pain in a compressive pathology.

Mouse models were used in this study because they allow direct comparison between a standardized contusion SCI model and the genetically defined *ttw*/*ttw* model of chronic degenerative compressive myelopathy, which recapitulates key pathological features of human disease. In addition, mice enable high-resolution spatiotemporal analyses of M/M activation across the spinal and supraspinal regions using well-established molecular tools. Although many neuroinflammatory signaling pathways involved in NeP are conserved across species, species-specific differences limit direct extrapolation of these findings to humans. Therefore, the present findings should be interpreted as providing mechanistic insight into conserved processes rather than direct clinical prediction, and further validation in complementary models and human studies is warranted.

**Figure 8:**
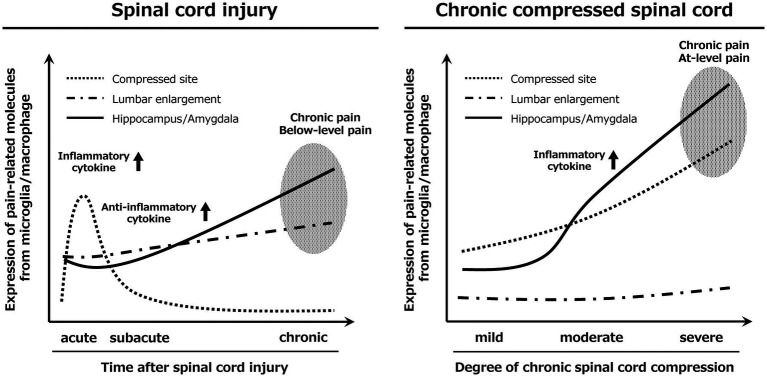
Schematic summary of microglial dynamics and neuroinflammation underlying neuropathic pain. In SCI, M/M activation and pain-related molecules localized to the lesion site acutely, shifted to the lumbar enlargement during the subacute-to-chronic phases, and subsequently involved the hippocampus and amygdala. In contrast, DCM showed progressive M/M activation at the compression site and in the limbic brain regions, with minimal lumbar involvement. Stronger hippocampal and amygdalar activation in DCM may underlie the refractory pain profile.

Several limitations of the present study should be acknowledged. The SCI and DCM models differed in mouse strain, genetic background, age, and anatomical level of spinal pathology (thoracic contusion at T9–T10 versus cervical compression at C1–C2), all of which are known to independently influence M/M density, inflammatory signaling dynamics, baseline cytokine expression, behavioral pain sensitivity, and neuroanatomical development. These differences may confound direct quantitative comparisons between the two models; therefore, the present study was designed to evaluate temporal and spatial pathological patterns within each model rather than to establish strict equivalence between SCI and DCM. In addition, the distinct spinal levels involved may differentially affect ascending and descending pathways, which is particularly relevant when interpreting supraspinal M/M-associated inflammatory changes in regions such as the hippocampus and amygdala. Pain behavior in this study was evaluated using stimulus-evoked mechanical and thermal assays, which mainly reflect sensory-discriminative pain. Spontaneous pain and affective–motivational components were not directly assessed, representing an important limitation, particularly given our focus on supraspinal regions such as the hippocampus and amygdala. Thus, M/M-associated inflammatory changes observed in these regions should be interpreted as a potential substrate for affective and ongoing pain rather than a direct behavioral correlate. With respect to inflammatory phenotype, the present cytokine analysis should be interpreted with caution. Although changes in TNF-α, IL-4, and IL-10 expression were observed in a model- and stage-dependent manner, the limited cytokine panel examined in this study did not permit the definitive classification of M/M polarization into classical M1 or M2 phenotypes. In particular, the absence of significant changes in IL-12 underscores that cytokine expression alone is insufficient to define polarization states. Accordingly, the present findings are best interpreted as reflecting shifts in pro- versus anti-inflammatory signaling rather than strict polarization. Finally, CD11b immunofluorescence at the magnification used does not allow detailed morphological characterization or definitive distinction between resident microglia and infiltrating macrophages, particularly at injured or compressed spinal levels. Accordingly, the observed changes reflect alterations in the abundance and signaling activity of the combined M/M population, and the term “activation” is used operationally to denote increased CD11b-positive cell density accompanied by enhanced pain-related signaling rather than morphology-defined activation states. In summary (Figure 8), the SCI model showed M/M activation and pain-related molecular expression at the lesion site in the acute phase, in the lumbar enlargement in the subacute phase, and in the hippocampus and amygdala in the chronic phase. These findings are consistent with clinical observations. In contrast, the DCM model revealed M/M activation and pain-related molecular expression at the compression site, hippocampus, and amygdala, with stronger brain M/M-associated inflammatory changes than those observed in the SCI model. Moderate compression elevated inflammatory cytokines and induced pain-related molecules in the thalamus and somatosensory cortex, whereas changes were absent in the lumbar enlargement. Clinically, these patterns explain the pain profiles of patients. Taken together, the findings show the need to suppress hippocampal and amygdalar M/M activation for NeP therapy, but the optimal timing of intervention differs among pathologies. In acute SCI, targeting anti-inflammatory and neuroprotective strategies during the acute-to-subacute phases (within about 2 weeks of injury) may be critical to prevent secondary injury. In contrast, in DCM, M/M activation in the spinal compression site and emotion-related brain circuits progresses in proportion to compression, suggesting that early therapeutic intervention should be considered as soon as upper limb neurological symptoms emerge, even in the absence of motor impairment. In both conditions, once M/M activation and pain-related molecular expression are established in the hippocampus and amygdala, NeP is likely to progress to a refractory and chronic state.

## Data Availability

The raw data supporting the conclusions of this article will be made available by the authors, without undue reservation.

## Supplementary material

SUPPLEMENTARY FIGURE S1 Representative merged CD11b/DAPI images of SCI (sham and 12 weeks after injury) **(A)** and DCM (ICR and 24-week-old ttw/ttw mice) **(B)** models at the lesion site and lumbar enlargement. Scale bars = 50 μm.

SUPPLEMENTARY FIGURE S2 Representative merged CD11b/DAPI images of SCI (12 weeks after injury) **(A)** and DCM (24-week-old ttw/ttw mice) **(B)** models in the brain. Scale bars = 50 μm.

SUPPLEMENTARY FIGURE S3 Representative single-channel images (p-p38, p-ERK1/2, and CD11b) with merged images in SCI (2 weeks after injury) **(A)** and DCM (24-week-old ttw/ttw mice) **(B)** models at the lesion site and lumbar enlargement. Scale bars = 50 μm.

SUPPLEMENTARY FIGURE S4 Representative single-channel images (p-p38, p-ERK1/2, and CD11b) with merged images in SCI (12 weeks after injury) **(A)** and DCM (24-week-old ttw/ttw mice) **(B)** models in the hippocampus and amygdala. Scale bars = 50 μm.
