# Correction: Differential microglial dynamics and neuroinflammation underlying neuropathic pain in the central nervous system: comparative insights from spinal cord injury and compressive myelopathy models

**DOI:** 10.3389/fncel.2026.1877129

**Published:** 2026-05-26

**Authors:** Arisa Kubota, Hideaki Nakajima, Kazuya Honjoh, Shuji Watanabe, Ai Takahashi, Akihiko Matsumine

**Affiliations:** Department of Orthopaedics and Rehabilitation Medicine, Faculty of Medical Sciences, University of Fukui, Fukui, Japan

**Keywords:** central nervous system, degenerative compressive myelopathy, microglia/macrophage, neuroinflammation, neuropathic pain, ossification of posterior longitudinal ligament, spinal cord, spinal cord injury

There was a mistake in Figure 7 as published. Upon carefully reviewing the final version, we noticed an error in the annotation of Figure 7.

Specifically, the labeling within the graph is incorrect. The correct annotation should read “Injured” or “Compressed” and “Lumbar” instead of the current version (TNF-α, IL-12, and IL-4, IL-10).

Importantly, this issue is limited solely to the figure annotation. The Figure Legend, main text, results, and conclusions of the article remain accurate and do not require any modification.

We kindly request that a correction be issued to replace the incorrect figure annotation with the correct version.

The corrected Figure 7 appears below.

**Figure 7:**
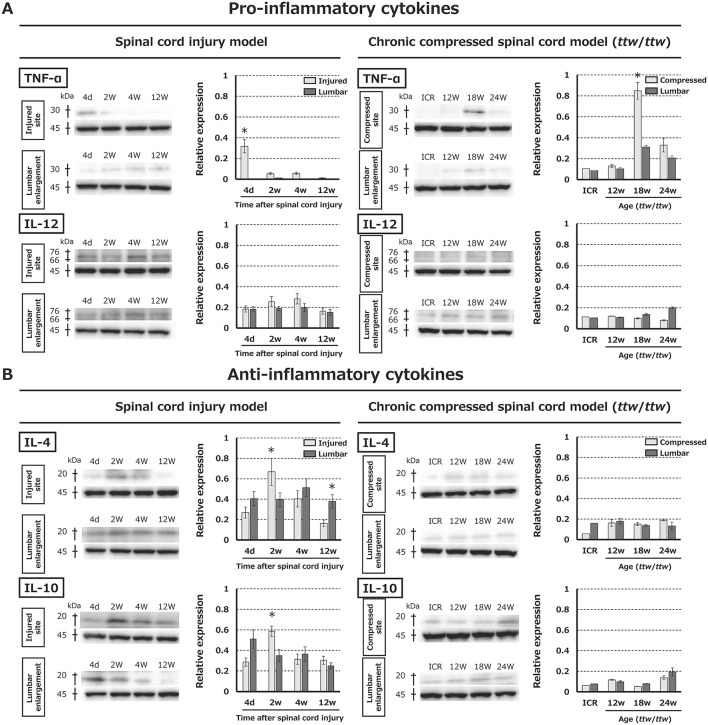
Cytokine expression in the spinal cord in the SCI and DCM models. **(A)** For pro-inflammatory cytokines, TNF-α peaked at 4 days post-injury in SCI and at 18 weeks in DCM, while IL-12 expression did not change significantly in either model. **(B)** Anti-inflammatory cytokines IL-4 and IL-10 peaked at 2 weeks post-injury in SCI, but showed no significant changes in DCM. ^*^p < 0.05.

The original version of this article has been updated.

