# Supplementary figures and images for: Differential microglial dynamics and neuroinflammation underlying neuropathic pain in the central nervous system: comparative insights from spinal cord injury and compressive myelopathy models
Source: Front Cell Neurosci. 2026 Jan 23;20:1769004. doi: 10.3389/fncel.2026.1769004 (PMC12875993; doi:10.3389/fncel.2026.1769004)

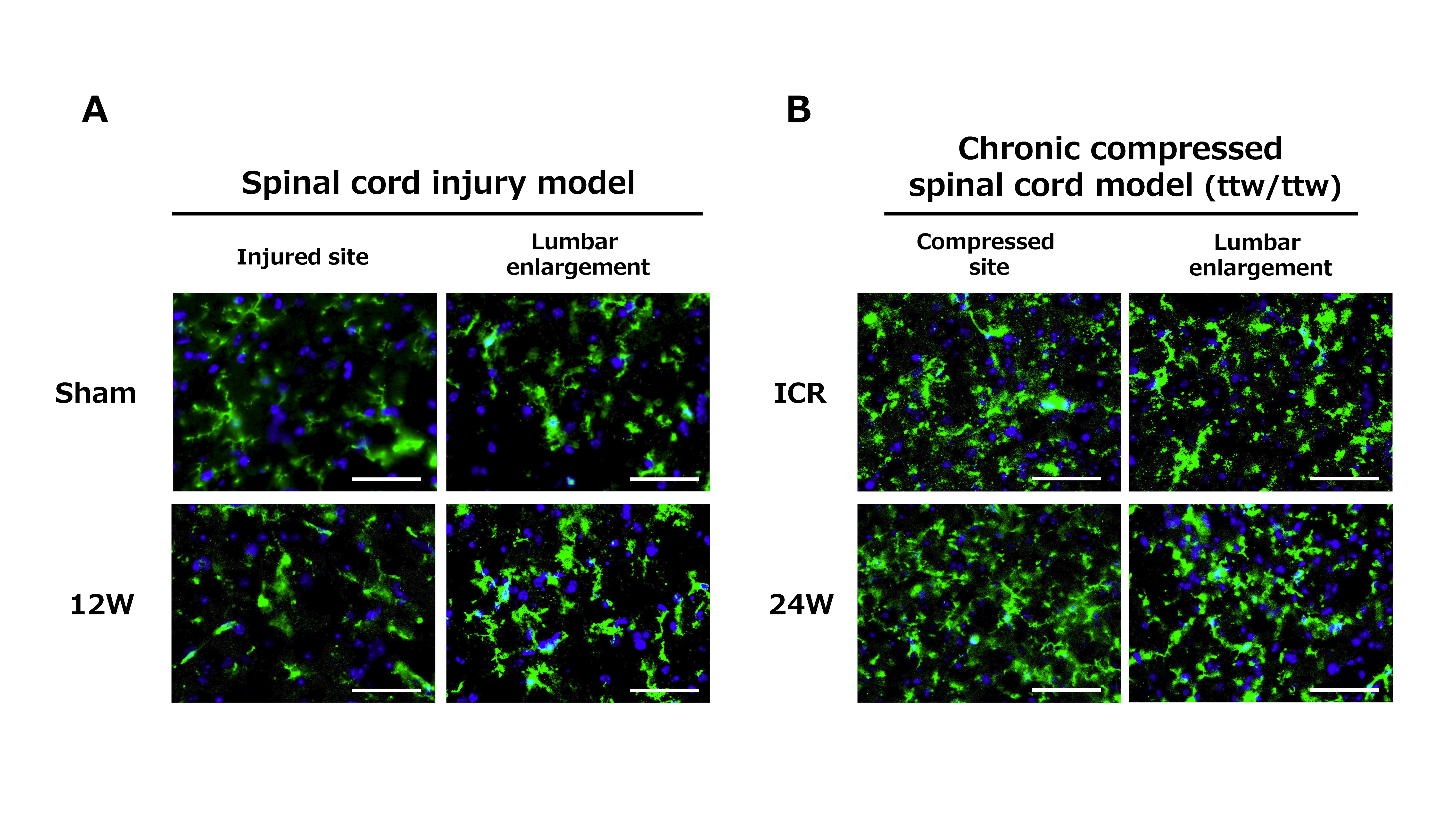

Supplement: SUPPLEMENTARY FIGURE S1 — Representative merged CD11b/DAPI images of SCI (sham and 12 weeks after injury) (A) and DCM (ICR and 24-week-old ttw/ttw mice) (B) models at the lesion site and lumbar enlargement. Scale bars = 50 μm. [file Image_1.TIF]

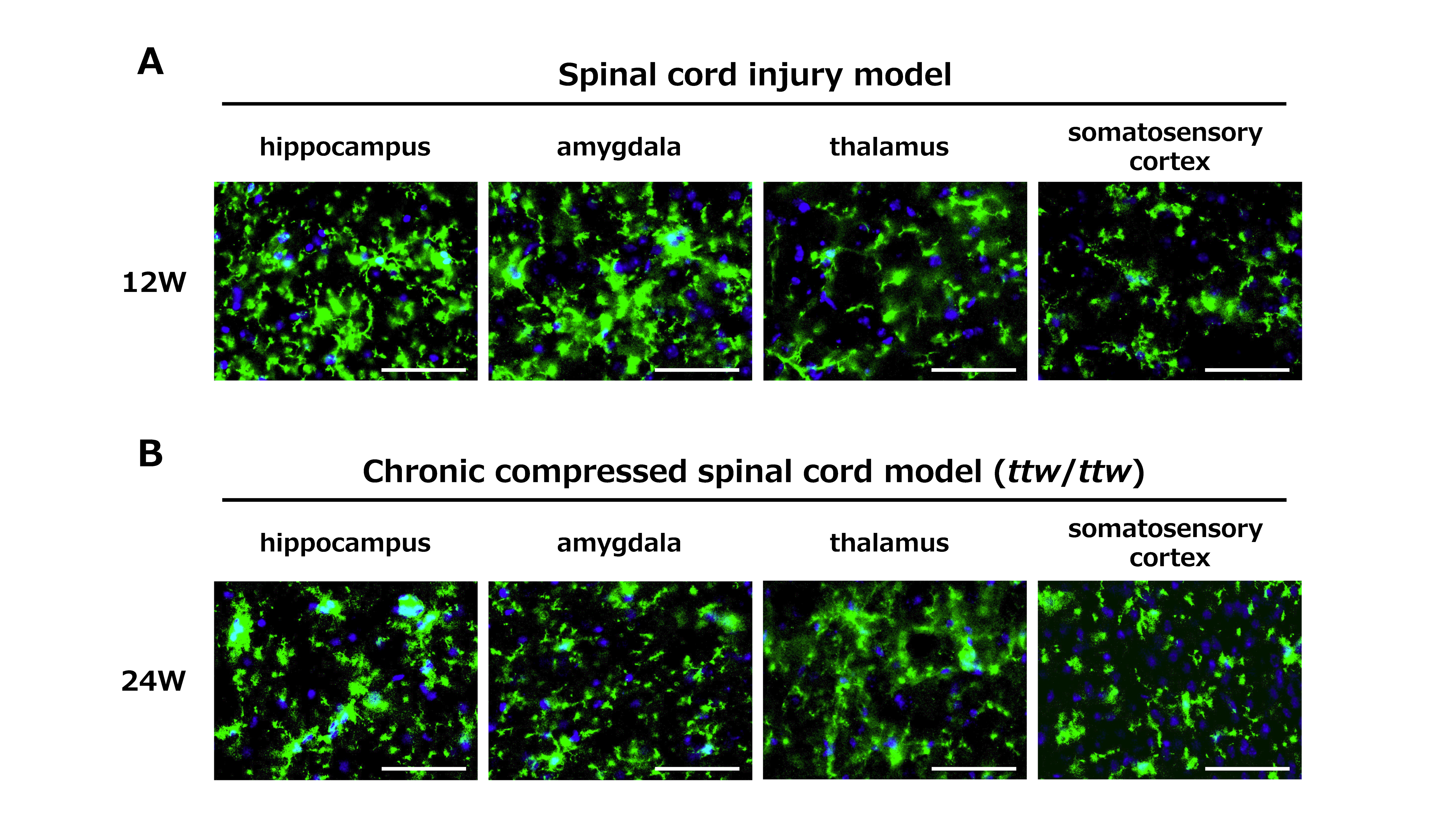

Supplement: SUPPLEMENTARY FIGURE S2 — Representative merged CD11b/DAPI images of SCI (12 weeks after injury) (A) and DCM (24-week-old ttw/ttw mice) (B) models in the brain. Scale bars = 50 μm. [file Image_2.TIF]

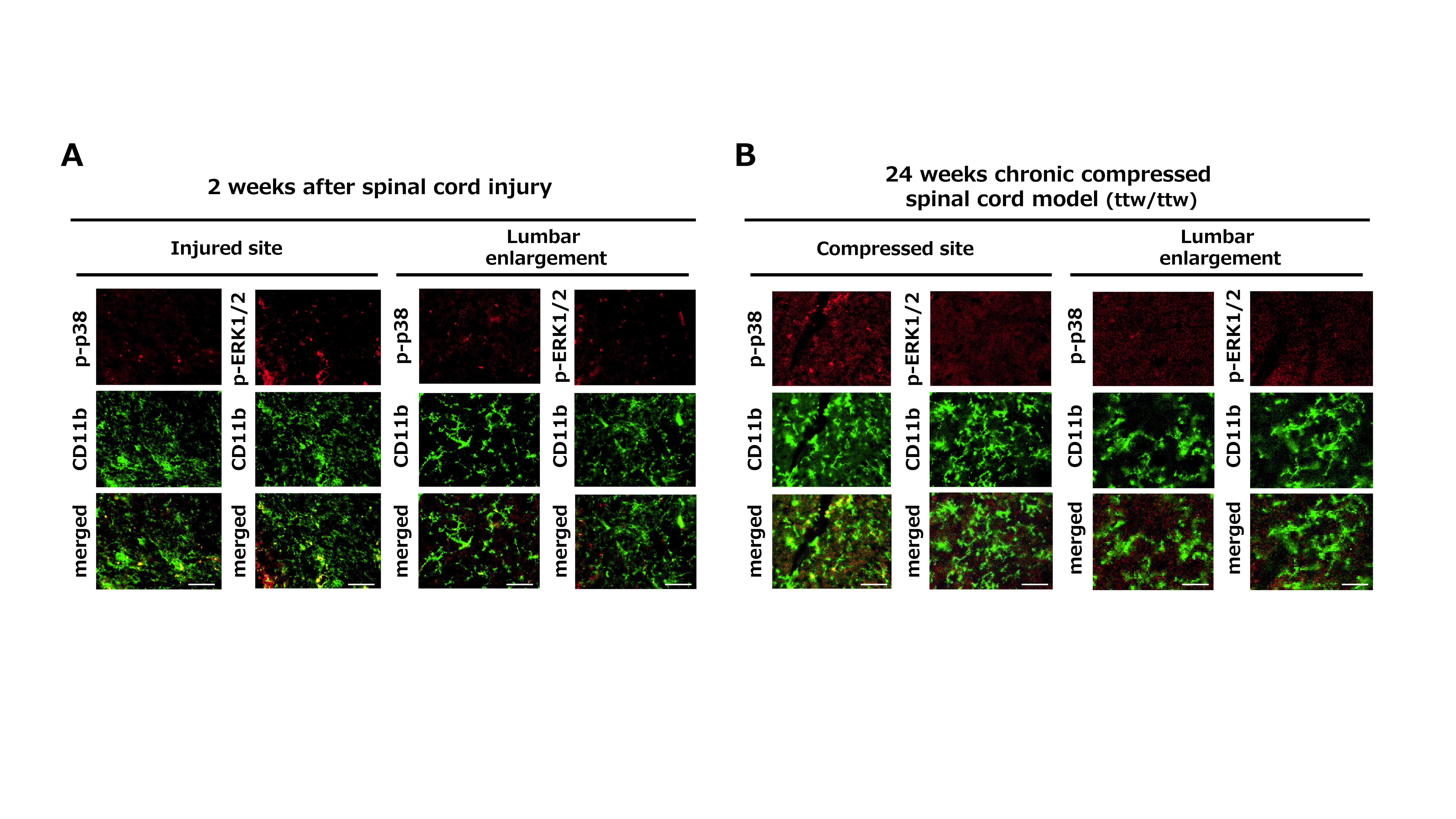

Supplement: SUPPLEMENTARY FIGURE S3 — Representative single-channel images (p-p38, p-ERK1/2, and CD11b) with merged images in SCI (2 weeks after injury) (A) and DCM (24-week-old ttw/ttw mice) (B) models at the lesion site and lumbar enlargement. Scale bars = 50 μm. [file Image_3.TIF]

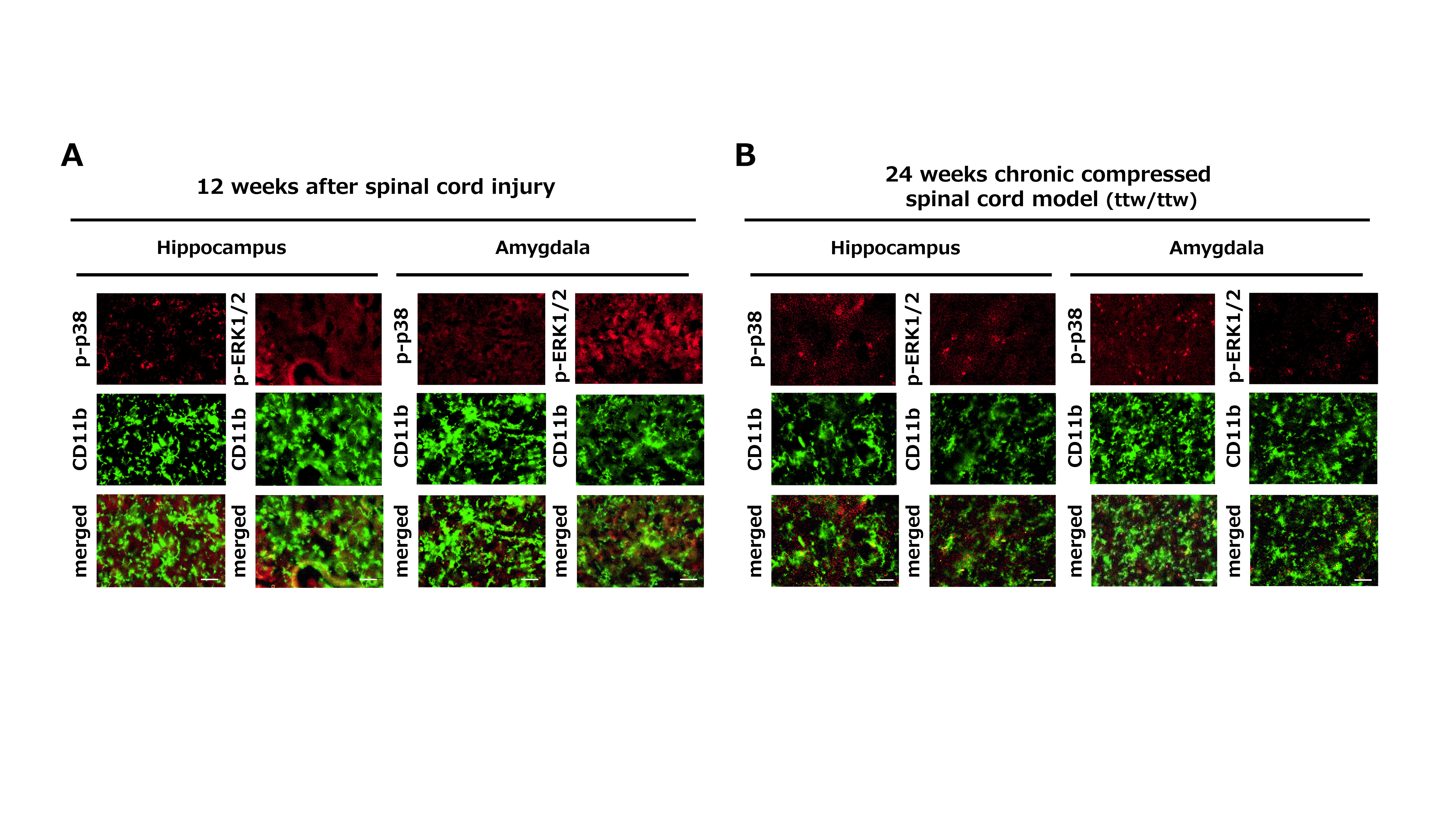

Supplement: SUPPLEMENTARY FIGURE S4 — Representative single-channel images (p-p38, p-ERK1/2, and CD11b) with merged images in SCI (12 weeks after injury) (A) and DCM (24-week-old ttw/ttw mice) (B) models in the hippocampus and amygdala. Scale bars = 50 μm. [file Image_4.TIF]
